# On the Discrepancy of Medical Opinions

**Published:** 1814-09

**Authors:** James Woodham

**Affiliations:** West Smithfield


*For the Medical and Physical Journal.*

*On the Discrepancy of Medical Opinions; by* Mr. WOODHAM.

WHEN we take a survey of the opinions of medical men, not only with respect to the theory, but, what is of the last importance, the treatment of diseases, we cannot fail to be struck with their discrepancy, but also with astonishment that men, from similar premises, should draw conclusions so directly opposite. It is a phenomenon in the history of the human mind, which would *à priori* be considered impossible. We need, however, only mention the opinions and practice of a few of the moderns, and those too of deserved reputation, to prove the truth of our assertion.

Cullen and Fordyce, names dear to most of the profession, maintain fever to be a disease of the whole system, recommend bleeding and purging in the most guarded manner, and rely for the cure principally on antimonials, to which they were both much attached. Dr. Clutterbuck and Dr. Beddoes consider fever as a symptom of organic inflammation. The former states this inflammation to be in the brain; the latter in some abdominal viscus. Both recommend free bleeding, generally and locally, together with active purgatives, which they regard as the chief remedies. Darwin and Currie, men of great talents, the former of whom was for many years in extensive practice, treated typhus with small doses of bark, opium, and wine; to which the latter added cold affusion. Dr. Parr, and Dr. Hamilton of Edinburgh, advise active purgatives only. Rush and his adherents assert that the yellow fever is not contagious, and a disease of high inflammation; while others of equal eminence maintain that it is contagious, and a disease of great debility. Fordyce and Dr. Haygarth recommend bark for the cure of acute rheumatism: the former says, metastasis used frequently to occur when he employed bleeding, but after he gave bark, which he did for some years previously to his death, he seldom lost a patient. Dr. Sutton, the latest writer on this disease, condemns the use of bark, advises bleeding freely, purgatives, refrigerants, and cold lotions to the joints. The generality of practitioners, in most pectoral complaints, advise advise the wearing of flannel, and recommend a temperature of a regular warmth, and which last has been much insisted on by Dr. Buxton;—Dr. Sutton before-mentioned, on the contrary, regards flannel as highly injurious, and orders cold lotions to be applied to the chest. Most surgeons consider the action of the capillary vessels to be increased in inflammation; Dr. Lubbock, Mr. Allen, and Dr. A. P. Wilson, say that is diminished; while Dr. Thompson, the Professor on Military Surgery at Edinburgh, maintains that it is sometimes increased and sometimes diminished. Sir James Earle, and the majority of surgeons, employ cold applications, or applications producing cold, in the inflammation consequent to burns; Mr. Kentish and some others use stimulating remedies, such as Ol. Terebinth mixed with Ceratum Resinæ. From the time when Cullen first published his Nosology, and Bell his Treatise on Ulcers, local diseases were for the most part, till within these few years, considered as requiring topical remedies only; Mr. Abernethy, a gentleman no less distinguished for his profound knowledge of anatomy than his great skill in surgery, believes them to have a constitutional origin, a disordered state of the digestive organs, and to the correction of which state the attention should be almost solely directed.

These are a few of the numerous discordancies existing among medical men. I have not enumerated them with a view to excite a spirit of scepticism or disgust, but to show young men about to enter on their practical career, the absolute necessity of thinking and observing for themselves.

*West Smithfield,* JAMES WOODHAM.

